# Reconstruction of Extensive Diaphragmatic Defects Using the Rectus Abdominis Muscle and Fascial Flap

**DOI:** 10.1055/a-1938-0763

**Published:** 2023-02-01

**Authors:** Shumpei Kato, Hisashi Sakuma, Takako Fujii, Ichiro Tanaka, Junichi Matsui

**Affiliations:** 1 Department of Plastic and Reconstructive Surgery, Ichikawa General Hospital, Tokyo Dental College, Chiba, Japan; 2 Department of Surgery, Ichikawa General Hospital, Tokyo Dental College, Chiba, Japan

**Keywords:** diaphragm, rectus abdominis muscle, fascial flap, reconstruction, case report

## Abstract

Diaphragmatic reconstruction is required for extensive diaphragmatic defects associated with tumor resection. Methods using artificial mesh and autologous tissues, such as pedicled flaps, have been reported predominantly for diaphragmatic reconstruction. We present the case of a 61-year-old woman who presented with a 14 × 13 × 12 cm tumor in the abdominal cavity of the upper left abdomen on computed tomography. The diaphragm defect measuring 12 × 7 cm that occurred during excision of the malignant tumor was reconstructed using the rectus abdominis muscle and fascial flap. The flap has vertical and horizontal vascular axes; therefore, blood flow is stable. It also has the advantage of increasing the range of motion and reducing twisting of the vascular pedicles. Fascial flap does not require processing such as thinning and can be used during suture fixation. This procedure, which has rarely been reported so far, has many advantages and may be a useful option for diaphragm reconstruction.

## Introduction

The diaphragm separates the thoracic and abdominal cavities and is an important muscle for respiratory function. However, the diaphragm might be resected during extended resection of lung cancer, mesothelioma, chest wall tumor, and soft-tissue sarcomas. 1 Depending on the size of the defect, diaphragmatic reconstruction may be required to minimize functional deterioration. Methods using artificial mesh and autologous tissues such as pedicled flaps have been reported primarily for diaphragmatic reconstruction. 2 We report a widespread diaphragmatic defect of 14 × 9 cm after tumor resection, which was reconstructed with a pedicle rectus abdominis muscle and fascial flap.

## Case

**Fig. 1:**
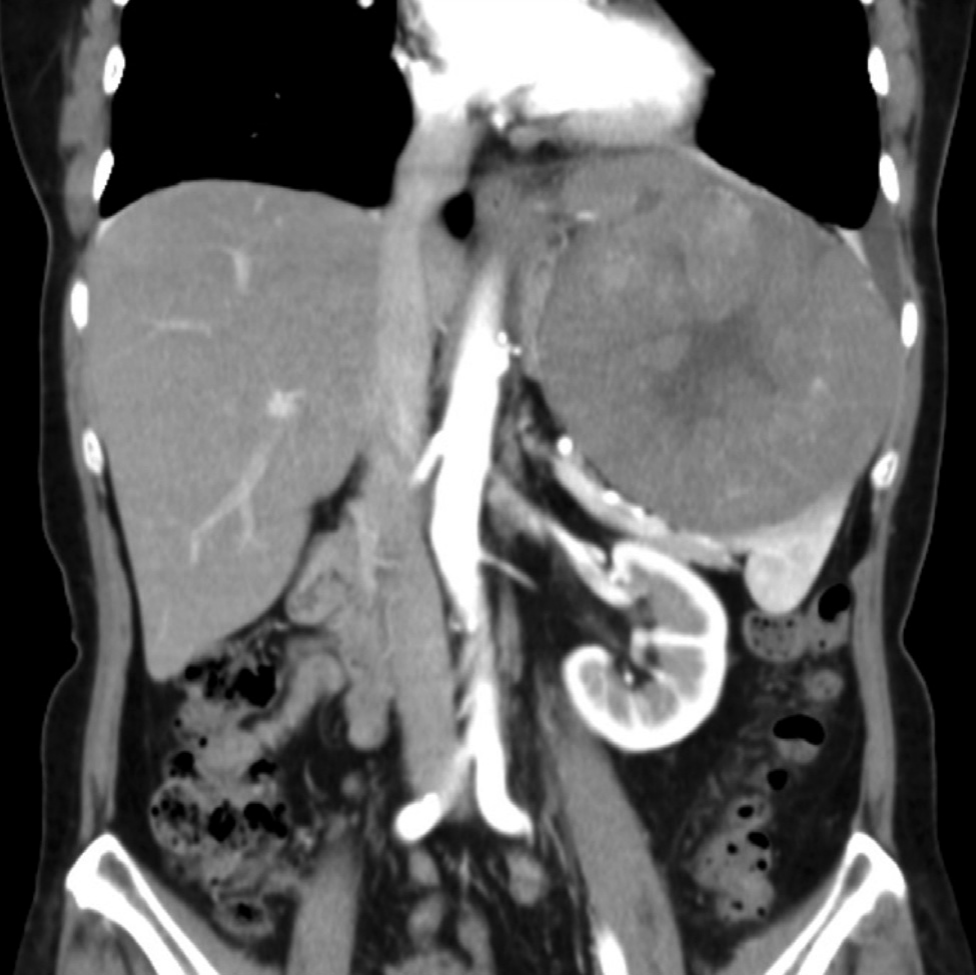
Computed tomography showed a 14 × 13 × 12 cm tumor in the upper left region of the abdominal cavity that was unevenly enhanced in the arterial phase.

**Fig. 2:**
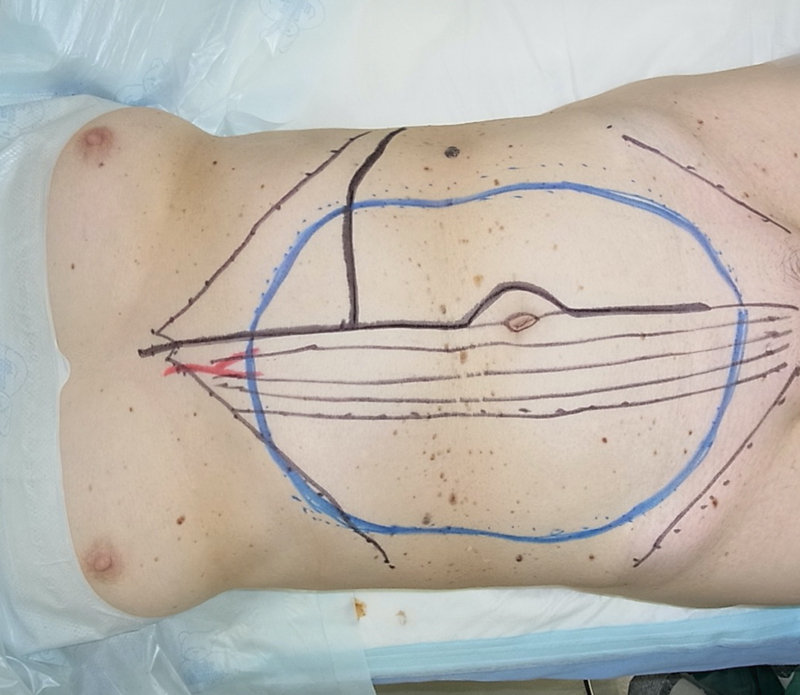
Patient is shown lying in supine position with midline abdominal and left hypochondrial incision markings.

**Fig. 3:**
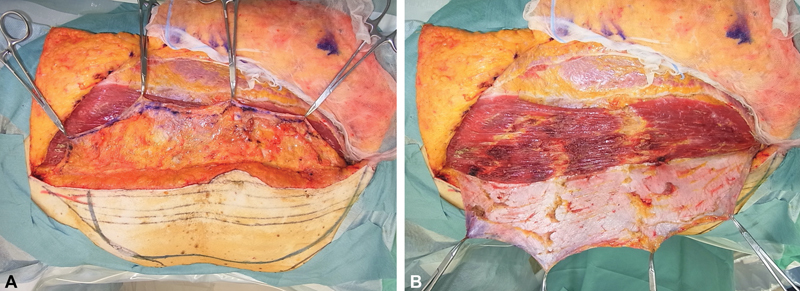
The left fascial flap up to 7 cm wide from the midline. ( **A** ) Front side of fascia. ( **B** ) Back side of fascia.

**Fig. 4:**
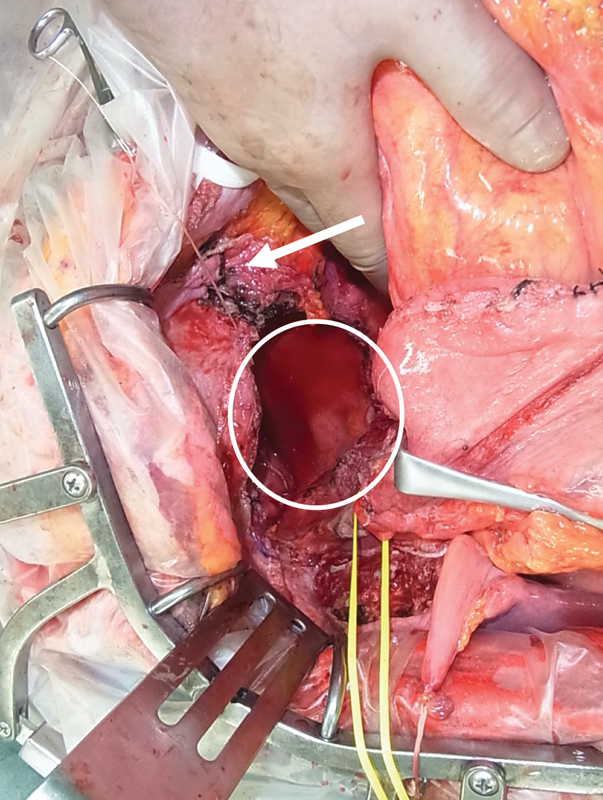
After tumor resection, the final defect size was ∼14 × 9 cm ( *white circle* ). Some defects could be repaired by suturing ( *white arrow* ).

**Fig. 5:**
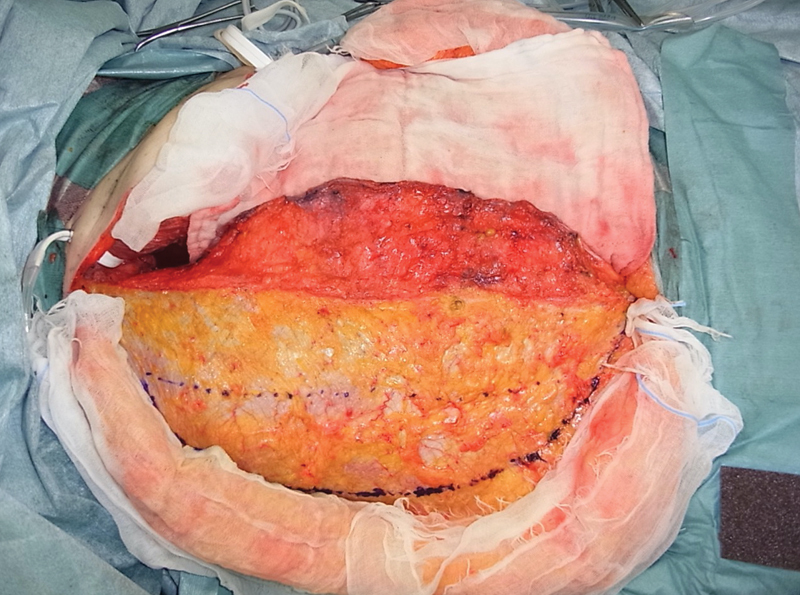
The right fascial flap. It was raised with a maximum width of 5 cm from the outer edge of the muscle.

**Fig. 6:**
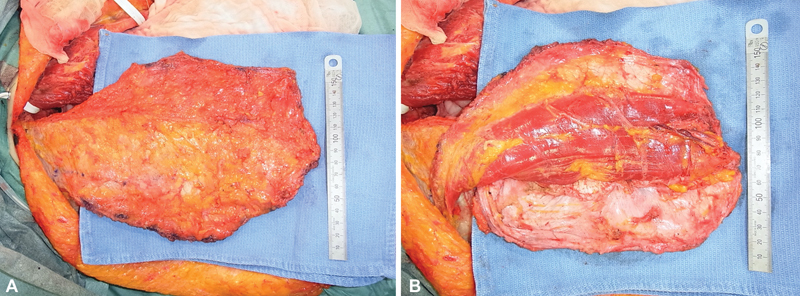
The pedicled rectus abdominis muscle and fascia flap was raised. ( **A** ) Front side of the flap. ( **B** ) Back side of the flap showing the right rectus abdominis muscle and the anterior sheath.

**Fig. 7:**
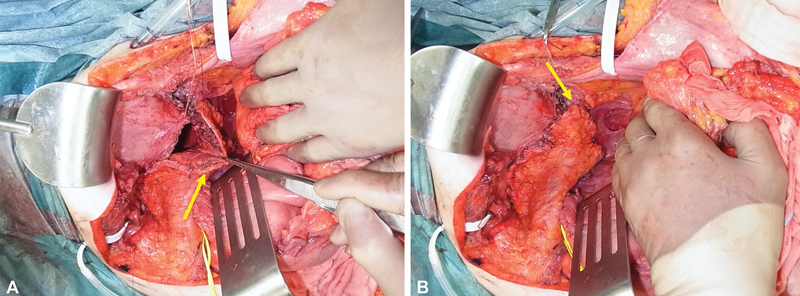
The rectus abdominis muscle and fascia flap was rotated counterclockwise to meet the diaphragmatic defect, with the muscle side placed on the thoracic cavity and the fascia side placed on the abdominal cavity. ( **A** ) The tip of the flap ( *yellow arrow* ). ( **B** ) The flap was sutured around the defect. The tip of the flap ( *yellow arrow* ).

**Fig. 8:**
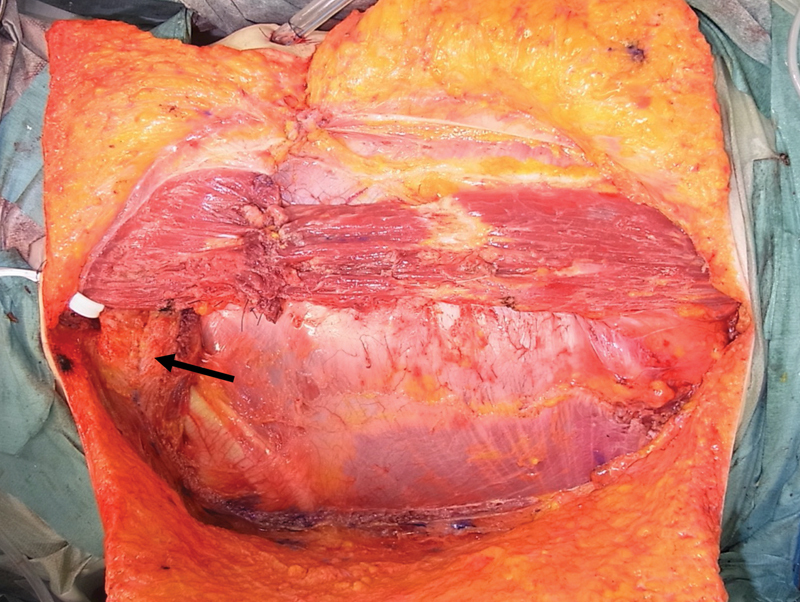
The abdominal wall was closed. The right and the left posterior sheaths, as well as the left rectus abdominis muscle can be visualized. On the upper right side, the base of the flap ( *black arrow* ) can be seen.

A 61-year-old woman was diagnosed with upper left abdominal pain. Computed tomography revealed a 14 × 13 × 12 cm tumor in the abdominal cavity of the upper left abdomen, which was unevenly contrast-enhanced in the arterial phase, suggesting a malignant tumor ( Fig. 1 ). The cranial side of the tumor was in contact with the diaphragm and lateral region of the liver. The right side of the tumor was in contact with the corpus, and the caudal side with the spleen. A biopsy was not performed because of possible tumor bleeding and intraperitoneal dissemination. Therefore, on suspicion of a nonepithelial splenic tumor with infiltration of the liver, diaphragm, and stomach, extended excision and diaphragm reconstruction were performed. The surgery was performed in the supine position with a midline abdominal incision and left hypochondrial incision ( Fig. 2 ). In this case, contamination due to intestinal complication resection and postoperative radiotherapy was expected. Therefore, we performed diaphragm reconstruction using the pedicled rectus abdominis muscle and fascial flap, which can be performed in the same surgical field without changing position. First, the left fascial flap, which was up to 7 cm wide from the midline, was raised while preserving the vascular network on the fascia as much as possible ( Fig. 3 ). We included the external oblique fascia, internal oblique fascia, and the anterior rectus abdominis sheath in the fascial flap. Subsequently, the abdomen was opened, the tumor was resected, and the lateral region of the liver, tail of the pancreas, spleen, pericardium, left diaphragm, and base of the left lung were resected. The left diaphragm had most of the defects; however, some of the defects could be sutured. The final defect size was approximately 14 × 9 cm ( Fig. 4 ). After the extent of the defect was confirmed, the right fascial flap was raised with a maximum width of 5 cm from the outer edge of the rectus abdominis muscle while preserving the vascular network on the fascia as much as possible ( Fig. 5 ). Continuity of the left fascial flap was maintained at the midline. Finally, the rectus abdominis muscle was raised in full length along the anterior sheath ( Fig. 6 ). The superior epigastric arteries and veins and the intercostal arteries and veins of the hypochondrium were used as vascular pedicles without scarifying the muscles. It moved counterclockwise to the diaphragm defect, the muscle side was placed on the thoracic cavity side, and the fascia side was placed on the abdominal cavity side ( Fig. 7 ). Because the flap raised for the defect size was sufficiently large, unnecessary parts were excised and sutured around the defect. The space between the esophageal hiatus and mediastinum was closed by suturing the flap to the tissues surrounding esophagus. The abdominal wall was closed by suturing the left and right posterior sheaths, together with the peritoneum ( Fig. 8 ). There was no posterior sheath on the right side of the arcuate line on the caudal side. Considering the risk of infection, it was closed only by the peritoneum, without mesh reinforcement. Postoperatively, inflammatory pleural effusion was observed momentarily but was spontaneously absorbed. With respect to postoperative ambulation, the patient started sitting on the first day after surgery and gradually started walking on the second day. The patient used an abdominal splint for 6 months after the surgery. Six years after the operation, there were no obvious respiratory disorders or complications such as diaphragmatic hernia or abdominal incisional hernia. The tumor was pathologically diagnosed as malignant lymphoma. Currently, sufficient lung dilation has been achieved, chemotherapy has been performed, and the patient's lymphoma is in remission.

## Discussion

The diaphragm separates the thoracic and abdominal cavities and is an important muscle for respiratory function. The flexibility of the diaphragm regulates pressure in the thoracic and abdominal cavities and has a significant effect on human physiology. Diaphragm also has passages such as major arteries, veins, nervous system, and esophagus. 1 The causes of diaphragmatic defects include complications associated with malignant tumor resection, congenital diaphragmatic hernias, and trauma. If a diaphragm defect occurs, the above function will be lost and reconstruction will be necessary. Malignant tumors that invade the diaphragm, such as lung cancer, mesothelioma, chest wall tumors, upper abdominal soft-tissue sarcomas, and metastatic tumors, are the most common causes. Most tumors are only partially resected with sufficient margins, but sarcomas may require complete resection of the diaphragm. 1 2

Diaphragmatic reconstruction, using an artificial mesh and reconstruction using a pedicled flap, such as a latissimus dorsi myocutaneous flap, has been reported. 2 Moreover, there have been many reports of reconstruction using artificial mesh. 1 It has the advantages of being minimally invasive, completing the surgical procedure in a short time period, and obtaining stable strength. However, because of the risk of infection, combined intestinal resection is contraindicated, and postoperative radiotherapy is being considered. 3 It has also been reported that mesh increases the risk of esophageal strictures, such as erosion of the esophageal lumen, development of fibrosis, and dysphagia. 4 5 Additionally, it is expensive and has the potential to cause adhesive ileus. Santillan-Doherty et al reported use of biological materials, but these are also at risk of infection and are expensive. 6 Some studies reported diaphragmatic reconstruction with fascia lata. 7 8 9 Not only in animal experiments but also in clinical cases, it has been reported that the fascia lata has new microvessels in the fibrous stroma, and wound healing was good due to the blood flow from the surrounding tissues. In animal experiments, it has been reported that the strength of the fascia lata collected from the reconstructed diaphragm is greater than that of the expanded polytetrafluoroethylene mesh. For this reason, the strength of this method of suturing with fascia instead of muscle may be guaranteed. However, its application in widespread defects and contaminated wounds remains a concern.

To compensate for these shortcomings, reconstruction with pedicled flaps such as the latissimus dorsi myocutaneous flap, abdominal wall muscle flap, and greater omentum flap has been reported. 1 3 Reconstruction with autologous tissue that maintains blood flow is suitable not only for infected wounds, but also for growing pediatric cases. Furthermore, there is a report that it can prevent paradoxical respiratory movement. 10 In particular, the latissimus dorsi myocutaneous flap is a surgical procedure that has been performed for many years in the field of respiratory surgery for empyema and intractable bronchial fistula. The advantage of this flap is that it can be raised in the same position when the tumor is removed in the lateral decubitus position. However, when performing tumor resection with the patient in the supine position, it is necessary to change the position. In addition, because the vascular pedicle is short, it may be necessary to sacrifice some of the feeding vessels to increase mobility and allow them to reach the deepest part of the thoracic cavity. Therefore, the procedure is complicated, and there is a concern that blood flow at the tip may become unstable. 11

As a technique similar to the flap, there is a report of diaphragm reconstruction using transverse rectus abdominis myocutaneous (TRAM) flap, which is often used in breast reconstruction in recent years. 2 Suturing of the dermal tissue is possible, and fixation is also obtained. Furthermore, the stability of blood flow is considered high because of the vascular network, such as the superficial fascia. However, suturing a bulky flap in the deep position requires proficiency. In addition, the weight of the tissue makes it impossible to reproduce the flexibility of the diaphragm, and it is doubtful whether it can regulate pressure in the thoracic and abdominal cavities.

The fascial flap has several advantages. It is possible to raise the flap with a stable blood flow during tumor resection in the supine position. Regarding blood flow, there have been several reports on the vascular network in the fasciocutaneous system. Cormac et al visualized the fascial vascular network of a cadaver under a microscope and evaluated the vascular axis and inner diameter of the vasculature. They contemplated that a vascular network develops around the fascia and that raising the muscle and fascia simultaneously leads to the stabilization of blood flow. 12 Another study reported fluorescent staining of the vascular network when the flap was raised. It has been reported that cases in which the flap was raised, including the anterior sheath of the rectus abdominis muscle, obtained wider fluorescent staining in the area of Zone IV. 13

We can reconstruct cases with extensive diaphragmatic defects, contamination with intestinal resection, and postoperative radiation therapy. Compared with the fascia lata, the fascial flap has vertical and horizontal vascular axes, so the blood flow is stable. It is also resistant to infectious wounds and is useful for reconstruction because better wound healing can be expected with the surrounding tissues. In addition, compared with reconstruction with reverse latissimus dorsi and reverse pectoralis major flaps, which are nourished by the perforator of the intercostal artery, it also has the advantage of increasing the range of motion and reducing twisting of the vascular pedicles. Compared with TRAM, the flap does not require processing such as thinning, and the deep position can be fixed without problems. In addition, because the fascia can be used during suture fixation, strong fixation is possible. The disadvantage is that the wide range of fascial flaps makes the abdominal wall fragile, particularly on the caudal side of the arcuate line.

For a widespread diaphragmatic defect after tumor incision, we performed reconstruction with the rectus abdominis muscle and fascial flap, which has been rarely reported. This surgical procedure, which has many advantages, may be a useful option for diaphragm reconstruction.
